# Relationship Between Ankylosing Spondylitis and Cerebrovascular Disorders: A Systematic Review

**DOI:** 10.5152/eurasianjmed.2024.23330

**Published:** 2024-06-01

**Authors:** Mohammad Mahdi Heidari, Azad Khaledi, Amir Mohammad Taravati, Ali Rastegar-Kashkouli, Farzaneh Moammer, Iman Saffari

**Affiliations:** 1 Department of Pediatrics, Kashan University of Medical Sciences Faculty of Medicine, Kashan, Iran; 2 Infectious Diseases Research Center, Kashan University of Medical Sciences, Kashan, Iran; 3 Department of Microbiology and Immunology, Kashan University of Medical Sciences School of Medicine, Kashan, Iran; 4 Isfahan University of Medical Sciences, Isfahan, Iran; 5 Isfahan University of Medical Sciences School of Medicine, Isfahan, Iran; 6 Student Research Committee, Guilan University of Medical Sciences School of Medicine, Rasht, Iran; 7 Department of Food Hygiene and Quality Control, Islamic Azad University Faculty of Veterinary Medicine Science and Research Branch, Tehran, Iran

**Keywords:** Ankylosing spondylitis, cerebrovascular disorders, risk factors, myocardial infarction

## Abstract

Cerebrovascular events are linked to ankylosing spondylitis. Accelerated atherosclerosis and endothelial dysfunction against a backdrop of inflammation have been widely blamed for the increased cerebrovascular risk in ankylosing spondylitis. The absence of a comprehensive review encouraged us to consider the link between ankylosing spondylitis and cerebrovascular diseases.

Web of Science, PubMed, Medline, Scopus, and EMBASE were searched to identify studies published from 2000 to June 10, 2023. All observational and cohort studies reporting myocardial infarction or stroke and considering classic cerebrovascular risk in ankylosing spondylitis patients and healthy controls were included.

Most of the included studies reported that the risk of cerebrovascular disorders was greater in ankylosing spondylitis than in the general population. Also, most studies showed that for both sexes, the prevalence of cardiovascular illnesses rose with age, and this trend was consistent across all subgroups of cardiovascular disorders. Also, most studies reported that the rate of cerebrovascular accidents in ankylosing spondylitis patients was higher than in control groups. Some studies reported that the risk of developing an ischemic stroke was higher in young patients with ankylosing spondylitis, while others did not.

Our systematic analysis found that most studies agreed that ankylosing spondylitis patients had a higher risk of cerebrovascular diseases than the general population. Still, this increased risk was influenced by several factors that need further research exploration.

**Main Points**

- In total, this review found that most studies agreed that ankylosing spondylitis patients had a higher risk of cerebrovascular diseases than the general population.
- Most studies showed that for both sexes, the prevalence of cardiovascular illnesses rose with age, and this trend was consistent across all subgroups of cardiovascular disorders.
- Most studies reported that the rate of cerebrovascular accidents in ankylosing spondylitis patients was higher than in control groups.

## Introduction

Cardiovascular diseases (CVD) include a wide range of diseases related to blood vessels and heart.^1,2^ Cardiovascular diseases kill many patients in the world.^3^ Cardiovascular illnesses kill more than 60 million years of life in European countries yearly, including early deaths in those under 70, and men have greater age-standardized rates of morbidity and mortality from CVD than women, especially in those under 70.^4^ Cardiovascular diseases in recent years have been increasing.^5^ It is well known that people with inflammatory rheumatic sicknesses have a higher chance of rising CVD.^6^

A lot of research showed that chronic systemic inflammation makes atherosclerosis more likely to happen and makes people more likely to get blood clots by interfering with normal blood clotting and causing a state of coagulation dysfunction.^7^ In this situation, inflammatory diseases like rheumatoid arthritis (RA) and ankylosing spondylitis (AS) have been connected to cerebral events.^8^ Augmented cardiovascular risk in AS has been connected to inflammation and its effects on atherosclerosis and endothelial function.^9^ Hypertension^9,10^ and metabolic syndrome^11,12^ as 2 cerebrovascular event (CVE) risk factors have a high prevalence in AS.

The 3E Initiative in Rheumatology states that AS is present if many of the following characteristics are present, in addition to persistent back pain of at least 3 months’ duration: sacroiliitis/spondylitis by imaging, alternating buttock pain, response to non-steroidal anti-inflammatory drugs (NSAIDs), inflammatory back pain, symptom onset before age 45, presence of confirmed acute anterior uveitis, presence of peripheral disease manifestations (arthritis, dactylitis, or enthesitis), presence of HLA-B27 positivity, and positive family history of disease.^13,14^

Nonsteroidal anti-inflammatory drugs are suggested as the first treatment.^15 16^ In contrast to their usage in other kinds of inflammatory arthritis, when they are taken just during flares, NSAIDs are commonly used long-term and continuously in AS. Furthermore, it is well recognized that various forms of NSAIDs have a varying risk of CVE.^17 18^ Cox-2 inhibition is responsible for the anti-inflammatory and analgesic benefits of NSAIDs, but it also increases hypertension and platelet activation in response to thromboxane, creating a net prothrombotic impact. It has been demonstrated via research on RA that TNFi is linked to a lower chance of CVE risk, most likely as a result of a less inflammatory load.^19^

Evidence in axial spondyloarthritis (SpA) patients is largely restricted to individuals with radiographic disease symptoms, i.e., AS, also known as radiographic axial spondyloarthritis (r-axSpA). Psoriatic arthritis (PsA) is also a source of proof in peripheral SpA cases.^20^

It is well-established that RA patients have a greater risk of cardiovascular death compared to the general population,^21^ but statistics for PsA are less clear. Nonetheless, an analysis conducted by Jamnitski et al^22^ reported higher rates of hypertension prevalence in patients with AS compared to those without AS. In the face of this, there was no rise in the occurrence of cerebrovascular disease/stroke in patients with AS compared to people without AS.^23^ A higher risk of ischemic heart disease has also been linked to AS, according to studies conducted previously.^11^

Although the findings of these studies are crucial, the authors’ attention was primarily directed toward the diagnosis of AS, and little is recognized around the connection between AS and cerebrovascular disorders. Since no recent systematic review has addressed this gap in knowledge, it encouraged us to consider the link between AS and cerebrovascular.

## Material and Methods

### Search Strategy

PubMed, and EMBASE was explored to recognize studies published from 2000 to June 10, 2023. All observational studies monitoring MI or stroke and all cohort studies seeing classic (blood glucose, blood pressure, lipid summary, metabolic syndrome, and body mass index (BMI)), and newer CV risk factors in AS people and healthy people were involved.

## Eligibility Criteria

### Inclusion Criteria

The studies published in English up to December 2022, case–control and observational studies reported the population of AS patients agreeing to the reliable standards,^24^ data containing the number of MIs or strokes, or the CV risk factor profile included.

### Exclusion Criteria

The article that we did not include in our study were case reports, commentary or discussion, and studies comprising &gt;5 patients, no AS patients, no information around CV risk factors or CV illnesses, no full text, no visible data (no standard deviation (SD) or no interquartile range)

### Risk of Bias

To avoid reporting and database bias in systematic reviews, we also evaluated unpublished reports and materials through manual search. Due to limiting the search of this study to a database such as Medline, the language bias has also been removed. In order to remove the multiple publication bias in this study, after detailed investigations, only 2 studies were found that examined the same population. Due to the different reporting of the results in them (Park et al^25^ stated the results in the field of MI and Lee et al^26^ in the field of CVA), both studies remained in the final evaluation. In order to carry out this study, the review of the sources included in related articles was not accepted, so citation bias will not have a place in this study. Considering that in some of the reviewed articles, the reports were limited to only one aspect and the researchers only reported outcomes with significant results, there was a possibility of outcome reporting bias in this study. A case that is less studied will be safe from having this bias. In this study, a manual search was also done, so the researchers chose the existence of gray literature bias at the cost of eliminating the language bias. Regarding time lag bias and media attention bias, these 2 biases are not evaluated in this review.

### Quality Assessment of Studies

As shown in Supplementary 1, based on the Critical Appraisal Tool for Systematic Reviews (CASP) checklist,^27^ we assessed the quality of the reports included. The questions in this checklist are specific for each type of study method; questions 1 and 2 help to quickly determine the quality of the study. If the first 2 questions are positive, the other questions are also checked. There are 3 options for each question. There is that “YES,” “NO,” or “can’t say” was recorded depending on the opinion of each of the authors. At the end the responsible author gave the final opinion about each question after discussing it with the author for each question.

## Synthesis of Results

Stroke incidence rates in our study are the number of strokes as a function of a follow-up period, and stroke rate ratios as the ratio of stroke incidence rate in the observed group (e.g., AS) over in the normal people.

Some studies did not provide the number of patient years observed for the control group and the number of strokes, but instead only provided the rate ratio together with a confidence interval (CI). As per the Cochrane Handbook,^28^ CI can be converted to the natural logarithms of rate ratios and standard errors may be combined across studies using the generic inverse variance method. We used this method to combine stroke risk for the age category pooled analysis and for stroke.

## Results

**Figure 1.**
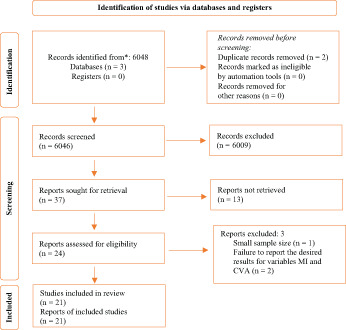
Preferred Reporting Items for Systematic Reviews and Meta-Analyses flow diagram for studies selection.

Figure 1 shows the studies that were finally retrieved and identified by the literature search. Of 6048 studies, 19 qualified studies were included after evaluations, and 2 via hand searching, for a total of 122 036 AS patients and 2 818 985 controls.

### Features of Studies

Of 21 publications, 1 was an observational study and 20 were cohort studies. Seventeen searches considered the prevalence of MI, and 14 studies judged stroke in AS patients. Ten of those considered both MI and stroke. Sixteen studies discussed covariates (diabetes, hypertension, hyperlipidemia, hypercholesterolemia, etc.) and presented data regarding the CV risk factors.

## Cardiovascular Events

### Myocardial Infarction

Of 16 studies, 6402 (n = 122 036) MIs were described. As presented in Table 1, 15 showed the MIs in the control groups (64 588 in 2 818  985). Szabo et al^29^ reported a high number of MI among the general population (GP) and AS patients; 19 113 in GP vs. 4127 MI in AS patients, in CV Definition 1, and in CV Definition 2; 8111 in GP vs. 2748 in AS. Their study was based on 2 definitions of cerebrovascular disease (when using CV Definition 1, the diagnosis of cardiovascular or cerebrovascular disease must be made using at least 1 applicable International Classification of Diseases, Ninth Revision (ICD-9) diagnostic code during the time period in question. When using CV Definition 2, the diagnosis must be made using at least 2 such codes). Most of the included studies showed that the prevalence of CVDs increased with increasing age for all CVD subgroups, and was similar for individuals of both sexes, but Essers et al^30^ expressed that in women, the risk of developing IHD was increased (hazard ratio (HR) 1.88, 95% CI 1.22-2.90), while the incidence rates were overall lower for women than men in the study conducted by Bengtsson et al.^31^ Although, in a study conducted by Walsh et al^32^ men have an increased risk of developing CVD compared to women, in contrast, Brophy et al^23^ indicated that there was no rise in MI in AS group compared to those without AS, in spite of the upper rate of hypertension.

### Cerebrovascular Accident 

In 14 studies (n = 12,853 patients), strokes were reported in AS patients. Twelve studies reported 44 021 strokes in control groups (Table 2). Except for Chou et al,^33^ other studies stated that the rate of CVA in AS patients was greater than control groups. In researches directed by Zöller et al,^34^ and Derakhshan et al,^35^ they only reported the incidence of CVA in AS patients and did not report it in control groups. In a study conducted by Zöller et al.^34^ 3477 patients (2416 men and 1061 women) had CVA, they concluded that admission to hospital, for many IMDs was related to increased risk of stroke. Also, Brophy et al.^23^ concluded that there was no increase in CVA rate in patients with AS compared to those without AS (40 in AS vs. 20 215 in control group). Similar to Essers et al,^30^ Bengtsson et al,^31^ Dong Hyun et al,^26^ Walsh et al,^32^ Trömmer et al,^36^ and Exarchou et al^10^ reported the same.

In a study conducted by Lin et al,^37^ the crude HR of ischemic stroke for AS group was 1.98 (95% CI, 1.20-3.29; *P* = .0079); age and sex were not included in the multiple regression analysis and of the 21 AS patients who developed stroke, 15 (71.4%) were male. The mean age of these 21 AS patients was 38.0 years (SD = 6.9), elder than that (31.3 years, SD = 7.6) of the remaining 4541 AS patients who did not develop stroke (*P *= .0001); they reported that the risk of developing ischemic stroke was higher in young patients with AS. Eriksson et al^38^ stated that there are 65 CVA in AS group vs. 148 CVA in the control group, also for stroke, the relative risks were 1.5 (1.1 to 2.0) in AS compared to the control group.

### Comparison of Hazard Ratio or Risk Ratio (95% Confidence Interval) for Myocardial Infarction

Based on data reported in Table 3, 6 studies reported HR or risk ratio (RR) (95% CI) for MI. The lowest HR was reported by Essers et al (HR: 0.90 (0.64-1.26) that adjusted to 0.76 (0.53-1.09). Other studies that reported HR for MI with 95% CI had similar values to the mentioned number. It should be noted that in the studies conducted by Szabo et al,^29^ values of standardized prevalence ratios (95% confidence intervals) were listed instead of HR and RR (1.25 (1.15-1.35). Additionally, Eriksson et al reported RR for MI, RR: 1.42 (1.08-1.86), adjusted RR: 1.3 (1.0-1.7).

### Comparison of Hazard Ratio or Risk Ratio or Odds Ratio (95% CI) for Cerebrovascular Accidents

As presented in Table 3, in 11 studies, authors reported HR or RR (95% CI) for CVA. The lowest HR was reported by Bengtsson et al, HR: 0.76 (0.64-0.89) that adjusted to 1.25 (1.06-1.48). Others reported HR for MI with 95% CI had similar values to the mentioned number, except for Keller et al, who reported a different and higher value than the others [2.3 (1.9–2.8)]. It should be noted that in Zoller et al’s study, values of standardized incidence ratio were reported instead of HR and RR (1.23 (1.01 - 1.48). It is noteworthy that Szabo et al^29^ reported OR in their study based on the age groups of the considered patients; in <50 years 1.45 (0.7-3.2), in 50-65 years 1.17 (0.91-1.50), and 65 years <1.12 (0.95-1.33). Hung et al also reported their results based on the follow-up periods of the patients; HR: 1.19 (0.89-1.58) and adjusted HR: 1.26 (0.94-1.68), in 3-year f/u HR: 1.16 (0.95-1.41) and adjusted HR: 1.14 (0.93-1.40), and 5-year f/u HR: 1.24 (1.05-1.46) and adjusted HR: 1.20 (1.02-1.42).

## Discussion

Cardiovascular disease is the key reason for mortality in many industrialized nations.^39^ Inflammatory rheumatic disorders are known to enhance CVD burden and risk^6^; As the report of previous studies in recent years showed this fact, but Bremander et al,^40^ Zoller et al,^34^ Buschiazzo et al,^41^ and Ahmed et al^42^ did not report it. First, these studies were mostly conducted before 2012, and until then the effective role of these factors in the mortality of patients with AS had not been proven. Since AS itself is chronic,^43^ all included studies have taken into account the necessity of conducting surveys over a prolonged period of time to accurately consider the effects on the mortality rate and the risk of MI and CVA.

Regarding the relationship between AS and the chance of MI, the RR or HR were analyzed in the studies we included. The data obtained from this study showed that the occurrence of MI in patients with AS increased compared to the control group, and this rate is at least 1.25 times.^29^ However, contrary to this fact, a study conducted by Essers et al^30^ reported this rate as HR: 0.90 (0.64-1.26), which they considered covariates such as adjusted for gender, age, CVD, renal failure, hypertension, BMI, alcohol use, smoking history, NSAIDs, antiplatelet, antihypertensive, antidiabetics, statin use, in the analysis with the multivariate Cox model method and adjusted hazard ratio expression, this rate was reported as adjusted HR: 0.76 (0.53-1.09) for MI. In their study, a large and statistically valid sample was used, but in contrast to prior studies which included both prevalent and incident patients, they only included those who had recently been diagnosed with AS. Misdiagnosis is a further problem due to the study’s wide definition of IHD which included various ischemic heart diseases and symptoms, including angina pectoris. Since many different medical issues may result in chest discomfort^44^ so, it’s conceivable to misdiagnose it. This should be noted, researchers looked at acute myocardial infarction as a more “reliable” marker for IHD, since it is identified with electrocardiogram and blood abnormalities. Also, Essers et al^30^ did not note the way to minimize of possible bias that may have happened every study.

Szabo et al^29^ reported a high number of MI among the GP and AS patients. However, Essers et al found that the occurrence of cardiovascular disorders did not increase with age for any subset of CVDs or between sexes,^30^ and expressed that in female patients, the risk of growing IHD was augmented (HR 1.88, 95%CI, 1.22-2.90), while the incidence rate was overall lower for women than men in a study carried out by Bengtsson et al^31^ Although, in a study conducted by Walsh et al,^32^ the risk of developing CVDs was higher in men compared to women.

Despite a higher prevalence of hypertension in patients with AS compared to those without AS, a finding that Brophy et al^23^ attribute to the use of NSAIDs, the risk of MI was not increased in the former group.

Young patients with a fresh diagnosis of AS have an increased chance of acquiring IHD, as shown by Huang et al.^45^ On the contrary, despite a historically high incidence rate of MI, this rate has reduced in recent years, as a trend seen by Eriksson et al,^38^ and Szabo et al^29^ showed that the point estimates were the uppermost in the earliest age. Although the incidence rate of MI was high in the past years, this rate declined from 2018 onward, but in a study conducted by Derakhshan et al^35^ from Iran, this rate was still high.

There is the same thing to investigate the relationship of CVA in AS patients, with the difference that Bengtsson et al^31^ considered other covariates, and they reported hazard ratio (HR) from 0.76 to 1.25. They also surveyed only patients diagnosed with incidental AS, although they covered a larger percentage of patients based on age groups (18-99 years); but for judging the reliability of their results, conducting a similar study in AS patients with the same age group in diagnostic subgroups (prevalent, incidental, etc.) is needed.

According to research conducted by Zöller et al,^34^ they determined that there was an elevated risk of ischemic or hemorrhagic stroke among those who had been hospitalized for several IMDs. Also, Brophy et al^23^ concluded that there was no increase in CVA in patients with AS compared to those without AS. Similar to Essers et al,^30^ Bengtsson et al,^31^ Dong Hyun et al,^26^ Walsh et al,^32^ Trömmer et al,^36^ and Exarchou et al^10^ reported the same. In a study conducted by Lin et al,^37^ of the 21 AS patients who had a stroke, 15 (71.4%) were men; however, neither sex nor age were included. They finally concluded that the risk of developing a stroke was higher in young patients with AS. Eriksson et al^38^ showed that the relative risks were 1.5 (1.1 to 2.0) for stroke in AS related to the control group. Generally, there was not enough data to support the idea that sex and age are effective in the frequency of CVA.

In the context of investigating the mortality of CVDs in rheumatological diseases; despite the fact that it is clear that RA patients have a higher CV mortality rate than the overall population,^21^ but the data for PsA are inconsistent where in 2013, Jamnitski et al^22^ performed a comprehensive study and found that patients with PsA had a higher death rate. Two studies included^46,47^ in our review revealed an increased standardized mortality ratio (SMR) of 1.4-1.6 for all mortality cases. On the contrary, a comprehensive perspective PsA cohort study carried out by Buckley et al^51^ from the UK, did not find a significant mortality increase [SMR 0.81 (95% CI 0.57-1.12)], although they reported that CV accounted for 38% of deaths,^48^ but what arose from the studies reviewed in this systematic review was that the comparison of mortality in patients with AS still requires more studies in this field, although the 2 studies^10,41^ which did this comparison definitely showed that the mortality rate is high in patients with AS.

Compared to age-matched healthy controls,^49^ individuals with AS have been shown to be more likely to be smokers and have a higher BMI. C-reactive protein, interleukin 6, and fibrinogen levels were all considerably higher in these individuals after controlling for smoking and body mass index.^50^ Although there was an increased incidence of conditions such hypertension, dyslipidemia, diabetes, obesity, and metabolic syndrome, these conditions alone cannot fully explain the higher risk shown in this cohort. Several noninvasive methods may be used to assess the extent to which chronic, systemic inflammation has contributed to the development of accelerated atherosclerosis (41). More studies are required to determine the impact of anti-inflammatory medication, particularly NSAIDs and biologics like TNFi, on the prevalence of CVD. Because risk factors are still underdiagnosed and undertreated, it is imperative that recommendations for care begin by emphasizing the elevated CV risk in AS and PsA. Then we can determine how to prevent these risk factors in the best possible way in such individuals.^51^

But, in the current studies, covariates such as diabetes, hypertension, hyperlipidemia, and hypercholesterolemia were considered, but the smoking status or paraclinical indicators were not evaluated. Considering the above, it is better to investigate the contradictory effect of covariates on the chance of MI and CVA in a study with a longer period and evaluate these covariates one by one to determine the reason for this discrepancy in the studies.

The main limitations of our review are: we only considered studies that were published in the English language, and the data may be contradictory to what is in reality. Also, in some cases, we encountered studies that required payment for downloading which we could not access due to our socioeconomic situation, although the authors of the studies were contacted in various ways (e-mail, ResearchGate, etc.), but we did not receive a response. Of course, it is necessary to state that first of its kind, the current research, we considered the correlation between MI and CVA in patients with AS.

Most studies included in the current review show that people with AS have a higher risk of cerebrovascular disorders than people in the general population. Nonetheless, this risk is influenced by diverse sources like smoking or non-clinical indicators, which need further investigation.

### Limitations

We only included the articles that we found online in the aforementioned databases, and we were not aware of unpublished studies and that only articles that were published in English were enrolled in this review.

## Figures and Tables

**Table 2.** Prevalence of Cerebrovascular Accident in Studies Included in the Present Review

Study	Publication Year	Number of CVA Patient	Number of CVA Control
Szabo et al	2011	1439	7554
Zoller et al	2012	3477	–
Brophy et al	2012	40	20215
Keller et al	2012	30/41	52/9
Chou et al	2013	2514	895
Lin et al	2014	21	53
Eriksson et al	2016	65	148
Essers et al	2016	44	317
Exarchou et al	2016	263	796
Bengtsson et al	2017	201	8954
Dong Hyun et al	2018	73	250
Walsh et al	2018	110	260
Derakhshan et al	2019	50	–
Trommer et al	2021	4526	4526

CVA, cerebrovascular accident.

**Table 3.** Comparison of Hazard Ratio or Risk Ratio (95% Confidence Interval) for Myocardial Infarction and Cerebrovascular Accident in Studies Included

First Author (y)	Country/Data Source	Participant/Age at Screening (y)	Follow-Up or Period (y)	Arthritis Female (%)	Patients (n)	Controls (n)	Covariates	HR or RR (95% CI) for MI	HR or RR or OR (95% CI) for Stroke	HR or RR (95% CI) for Mortality
Peters et al/2010	Netherland	AS/50-73	2007	26.4	383	75 333	–	HR: 3.75 (2.30-6.12)	–	–
Szabo et al/2011	Canada	AS/≥20	1996-2006	43.9	8616	50 699	Congestive heart failure, valvular (aortic or nonaortic) heart disease, ischemic heart disease, cerebrovascular disorder	1.25 (1.15-1.35)^1^	OR: 50 years 1.45 (0.7-3.2) 50-65 years 1.17 (0.91-1.50) >––65 years 1.12 (0.95-1.33)	–
BremandER et al/2011	Sweden	AS/≥20	2004-2007	32.8	935	761 210	–	–	–	1.45 (0.39-3.71)
Zoller et al /2012	Sweden/several national Swedish data registers (reviewed by Rosen and Hakulinen	AS/–	1987-2008	–	3477	216 291	–	–	^2^	–
Brophy et al/2012	Wales/Health Information Research Unit	AS/20-49	1999-2010	24.1	1686	1 206 621	Diabetes, hypertension, hyperlipidemia, hypercholesterolemia	HR: 1.28 (0.93- 1.74)	HR: 1.0 (0.73 -1.39)	–
Keller et al/2013	Taiwan/National Health Insurance Database	AS/≥40	2001-2005	37.3	1479	5916	Chronic lower, respiratory diseases, type 2 diabetes mellitus, hypertension, hyperlipidemia, renal disease, coronary heart disease, atrial fibrillation, income, and urbanization	HR: 1.34 (1.12-1.60)	HR: 2.3 (1.9–2.8)	-
Huang et al/2013	Taiwan/ National Health Insurance Database	AS/18-45	2000-2003	24.2	4794	23 970	Hypertension, diabetes mellitus, hyperlipidemia	1.47 (1.13 -1.92)	–	–
Chou et al/2013	Taiwan/ National Health Insurance Database	AS/≥18	2000-2009	–	6262	25 048	Hypertension, diabetes mellitus, hyperlipidemia, stroke, and peripheral vascular diseases	Adjusted HR: 1.36 (1.16-1.59)	HR: 1.49 (1.38-1.62)	–
Lin et al/2014	Taiwan/ National Health Insurance Database	AS/18-45	2000-2003	26.2	4562	22 810	Diabetes mellitus, hypertension, dyslipidemia, coronary heart disease	–	1.98 (1.20-3.29)	–
Ahmed et al/2016	UK	Ankylosing spondylitis, psoriatic arthritis, psoriasis	1999-2009	12.8	94	376	Hypertension, ischemic heart disease, hyperlipidemia, and diabetes mellitus	–	–	–
Eriksson et al /2016	Sweden/National patient register	Prevalent AS/≥18	2007-2012	32	5358	25 006	Age, gender, venous thromboembolism, COPD, diabetes, malignancy	RR: 1.42 (1.08-1.86) Adjusted RR: 1.3 (1.0-1.7)	RR: 1.62 (1.22-2.15) Adjusted RR: 1.5 (1.1-2.0)	–
Essers et al/2016	UK/Clinical Practice Research Datalink	Incidental AS/≥16	1987-2012	29.5	3809	26 197	Adjusted for age, gender, CVD, hypertension, renal failure, BMI, smoking history, alcohol use, NSAIDs, antihypertensive, antiplatelet, antidiabetics, statin use	HR: 0.90 (0.64-1.26) Adjusted HR: 0.76 (0.53-1.09) for MI	–	–
Exarchou et al /2016	Sweden/ NPR and census register	Incidental AS/≥18	2006-2012	34.5	8600	40 460	Age, gender, education, CVD, diabetes, infection, malignancy, chronic pulmonary disease, joint surgery, NSAIDs, glucocorticoids, sDMARD and TNF inhibitor use	–	–	HR: 1.52 (1.38-1.68) Adjusted HR: 1.6 (1.44-1.77)
Buschiazzo et al/2016	Argentina	AS/34-60	2000-2010	24.4	127	–	–	–	–	–
Hung et al/2016	Taipei	AS/≥40	2000-2005	51.8	537	2685	Adjusted for age, gender, income, region, hyperlipidemia	–	1 year f/u HR: 1.19 (0.89-1.58) Adjusted HR: 1.26 (0.94-1.68) 3 year f/u HR: 1.16 (0.95-1.41) Adjusted HR: 1.14 (0.93-1.40) 5 year f/u HR: 1.24 (1.05-1.46) Adjusted HR: 1.20 (1.02-1.42)	–
Bengtsson et al/ 2017	Sweden/National patient register	Incidental AS/18-99 Incidental uSpA/18-99 Incidental PsA/18-99	2006-2012	31.9 55.2 55.1	6448 5190 16 063	266 435	Age, gender, venous thromboembolism, diabetes, COPD, atrial fibrillation or flutter, another atherosclerotic disease		HR: 0.76 (0.64-0.89) Adjusted HR: 1.25 (1.06-1.48) Adjusted HR: 1.16 (0.91-1.47) HR: 0.96 (0.87-1.05) HR: 1.34 (1.22-1.48) for stroke	-
Dong Hyun et al/2018	Among the total population of the republic of Korea	AS/20-65	2010-2014	27.46	12 988	64 940	–	–	HR: 1.46 (1.13, 1.90)	–
Park et al /2018	Korea/ National Health Insurance Service	Incidental AS/≥20	2010-2015	27.46	12 988	64 940	Adjusted for age, gender, income, hypertension, diabetes, dyslipidemia	HR: 1.97 (1.47-2.65) Adjusted HR: 1.81 (1.34-2.34)	–	–
Lee et al/2018	Australian Rheumatology Association Database	Incidental AS/≥20	2001-2015	66.6	561	–	Adjusted for age, gender, hypertension, diabetes, dyslipidemia	–	–	–
Walsh et al/ 2018	USA	AS/≥18	2012-2014	39.5	6679	19 951	Adjusted for age, gender, hypertension, diabetes, dyslipidemia	HR: 1.41 (1.28-1.55)	HR: 1.41 (1.28-1.55)	–
Derakhshan et al/2019	22 participating countries throughout 5 continents (Africa, Asia, Europe, North and South America)	AS/18-100	5 years	35.1	2547	3923	Ever diagnosis of HTN, ischemic heart disease, stroke, diabetes mellitus, and dyslipidemia.	–	OR: 1.757 (0.796-3.881)	–
Trommer et al/2021	Germany	Incidental AS/≥18	2000-2015	65	29 106	29 106	Adjusted for age, gender, vascular risk factors (hypertension, diabetes, dyslipidemia)	–	HR: 1.41 (0.99-2.00)	–

AS, ankylosing spondylitis; BMI, body mass index; CI, confidence interval; CVD, cardiovascular diseases; HR, hazard ratio; NSAID, nonsteroidal anti-inflammatory drug; OR, odds ratio; RR, risk ratio.

COPD, Chronic obstructive pulmonary disease; HTN, Hypertension; NPQ, National Professional Qualifications; DMARDs, Disease-modifying antirheumatic drugs.

**Table:** **Table 1.** Prevalence of Myocardial Infarction in Studies Included in the Present Review

First Author (y)	Publication Year	Number of MI Control		Number of MI Patient		References
Szabo et al	2011	CV Definition 1	CV Definition 2	CV Definition 1	CV Definition 2	^29^
		19113	8111	4127	2748	
Bremander et al	2011	20		33		^40^
Zoller et al	2012	–		–		^34^
Brophy et al	2012	14 783		40		^23^
Keller et al	2013	517		171		^52^
Huang et al	2013	253		70		^41^
Chou et al	2013	221		584		^33^
Lin et al	2014	171		61		^37^
Ahmed et al	2016	36		10		^42^
Eriksson et al	2016	412		164		^38^
Essers et al	2016	354		69		^30^
Exarchou et al	2016	1488		556		^10^
Buschiazzo et al	2016	–		5		^41^
Hung et al	2016	–		–		^53^
Bengtsson et al	2017	8714		256		^31^
Dong Hyun et al	2018	250		–		^26^
Park et al	2018	157		62		^25^
Lee et al	2018	–		–		^54^
Walsh et al	2018	177		84		^32^
Derakhshan et al	2019	–		102		^35^
Trommer et al	2021	–		–		^36^

CV Definition 1, primary definition, requiring that the diagnosis of the cardiovascular or cerebrovascular disease be made according to at least 1 relevant International Classification of Diseases, Ninth Revision (ICD-9) diagnostic code during the time period; CV Definition 2, secondary definition, requiring that the diagnosis of the cardiovascular or cerebrovascular disease be made according to at least 2 relevant ICD-9 diagnostic codes during the time period.

## References

[1] Joseph P Leong D McKee M , et al. Reducing the global burden of cardiovascular disease, part 1: the epidemiology and risk factors. Circ Res. 2017;121(6):677 694. (10.1161/CIRCRESAHA.117.308903) 28860318

[2] Deaton C Froelicher ES Wu LH Ho C Shishani K Jaarsma T . The global burden of cardiovascular disease. Eur J Cardiovasc Nurs. 2011;10(2):S5 S13. 21762852 10.1016/S1474-5151(11)00111-3

[3] Townsend N Kazakiewicz D Lucy Wright F , et al. Epidemiology of cardiovascular disease in Europe. Nat Rev Cardiol. 2022;19(2):133 143. (10.1038/s41569-021-00607-3) 34497402

[4] Kim HC . Epidemiology of cardiovascular disease and its risk factors in Korea. Glob Health Med. 2021;3(3):134 141. (10.35772/ghm.2021.01008) 34250288 PMC8239378

[5] Ding X Wang X Wu J Zhang M Cui M . Triglyceride–glucose index and the incidence of atherosclerotic cardiovascular diseases: a meta-analysis of cohort studies. Cardiovasc Diabetol. 2021;20(1):76. (10.1186/s12933-021-01268-9) 33812373 PMC8019501

[6] Buleu F Sirbu E Caraba A Dragan S . Heart involvement in inflammatory rheumatic diseases: a systematic literature review. Medicina (Kaunas). 2019;55(6):249. (10.3390/medicina55060249) 31174287 PMC6632037

[7] Aksu K Donmez A Keser G . Inflammation-induced thrombosis: mechanisms, disease associations and management. Curr Pharm Des. 2012;18(11):1478 1493. (10.2174/138161212799504731) 22364132

[8] Tiosano S Yavne Y Gendelman O , et al. Stroke among rheumatoid arthritis patients: does age matter? A real-life study. Neuroepidemiology. 2017;49(3-4):99 105. (10.1159/000481992) 29136635

[9] Kelty E Ognjenovic M Raymond WD , et al. Mortality rates in patients with ankylosing spondylitis with and without extraarticular manifestations and comorbidities: A retrospective cohort study. J Rheumatol. 2022;49(7):688 693. (10.3899/jrheum.210909) 35428706

[10] Exarchou S Lie E Lindström U , et al. Mortality in ankylosing spondylitis: results from a nationwide population-based study. Ann Rheum Dis. 2016;75(8):1466 1472. (10.1136/annrheumdis-2015-207688) 26338036

[11] Mathieu S Gossec L Dougados M Soubrier M . Cardiovascular profile in ankylosing spondylitis: a systematic review and meta‐analysis. Arthritis Care Res. 2011;63(4):557 563. (10.1002/acr.20364) 20890982

[12] Kim J-W Yoon JS Park S Kim H . Kim by, Lee H, et al. Risk of cardiovascular disease associated with long-term use of non-steroidal anti-inflammatory drugs in ankylosing spondylitis. Rheumatology. 2024:kead684. 10.1093/rheumatology/kead684 38216768

[13] Sidiropoulos PI Hatemi G Song IH , et al. Evidence-based recommendations for the management of ankylosing spondylitis: systematic literature search of the 3E Initiative in Rheumatology involving a broad panel of experts and practising rheumatologists. Rheumatology (Oxford). 2008;47(3):355 361. (10.1093/rheumatology/kem348) 18276738

[14] Mansury D Ghazvini K Amel Jamehdar SA , et al. Increasing cellular immune response in liposomal formulations of DOTAP encapsulated by fusion protein Hspx, PPE44, and Esxv, as a potential tuberculosis vaccine candidate. Rep Biochem Mol Biol. 2019;7(2):156 166. 30805395 PMC6374059

[15] Ward MM Deodhar A Gensler LS , et al. 2019 update of the American College of Rheumatology/Spondylitis Association of America/Spondyloarthritis Research and Treatment Network Recommendations for the Treatment of Ankylosing Spondylitis and Nonradiographic Axial Spondyloarthritis. Arthritis Care Res. Spondylitis Association of America. 2019;71(10):1285 1299. (10.1002/acr.24025) PMC6764857 31436026

[16] Sertdemir AL Şahin AT Duran M , et al. Association between syndecan-4 and subclinical atherosclerosis in ankylosing spondylitis. Medicine. 2024;103(3):e37019. (10.1097/MD.0000000000037019) 38241528 PMC10798725

[17] Coxib and traditional NSAID Trialists ’ (CNT) Collaboration, Bhala N Emberson J , et al. Vascular and upper gastrointestinal effects of non-steroidal anti-inflammatory drugs: meta-analyses of individual participant data from randomised trials. Lancet. 2013;382(9894):769 779. (10.1016/S0140-6736(13)60900-9) 23726390 PMC3778977

[18] Dubreuil M Louie-Gao Q Peloquin CE Choi HK Zhang Y Neogi T . Risk of myocardial infarction with use of selected non-steroidal anti-inflammatory drugs in patients with spondyloarthritis and osteoarthritis. Ann Rheum Dis. 2018;77(8):1137 1142. (10.1136/annrheumdis-2018-213089) 29674321 PMC6045423

[19] Roubille C Richer V Starnino T , et al. The effects of tumour necrosis factor inhibitors, methotrexate, non-steroidal anti-inflammatory drugs and corticosteroids on cardiovascular events in rheumatoid arthritis, psoriasis and psoriatic arthritis: a systematic review and meta-analysis. Ann Rheum Dis. 2015;74(3):480 489. (10.1136/annrheumdis-2014-206624) 25561362 PMC4345910

[20] Pina Vegas L Le Corvoisier P Penso L Paul M Sbidian E Claudepierre P . Risk of major adverse cardiovascular events in patients initiating biologics/apremilast for psoriatic arthritis: a nationwide cohort study. Rheumatology (Oxford). 2022;61(4):1589 1599. (10.1093/rheumatology/keab522) 34244706 PMC8996783

[21] Castañeda S Nurmohamed MT González-Gay MA . Cardiovascular disease in inflammatory rheumatic diseases. Best Pract Res Clin Rheumatol. 2016;30(5):851 869. (10.1016/j.berh.2016.10.006) 27964792

[22] Jamnitski A Symmons D Peters MJ Sattar N McInnes I Nurmohamed MT . Cardiovascular comorbidities in patients with psoriatic arthritis: a systematic review. Ann Rheum Dis. 2013;72(2):211 216. (10.1136/annrheumdis-2011-201194) 22532629

[23] Brophy S Cooksey R Atkinson M , et al., eds. No increased rate of acute myocardial infarction or stroke among patients with ankylosing spondylitis—a retrospective cohort study using routine data. Semin Arthritis Rheum. Elsevier; Amsterdam. 2012;42(2):140 145. (10.1016/j.semarthrit.2012.02.008) 22494565

[24] Braun J Van der Heijde D Dougados M , et al. Staging of patients with ankylosing spondylitis: a preliminary proposal. Ann Rheum Dis. 2002;61(suppl 3):iii9 ii23. (10.1136/ard.61.suppl_3.iii19) 12381507 PMC1766734

[25] Park CJ Choi YJ Kim JG , et al. Association of acute myocardial infarction with ankylosing spondylitis: a nationwide longitudinal cohort study. J Clin Neurosci. 2018;56:34 37. (10.1016/j.jocn.2018.08.008) 30131197

[26] Lee DH Choi YJ Han IB , et al. Association of ischemic stroke with ankylosing spondylitis: a nationwide longitudinal cohort study. Acta Neurochir. 2018;160(5):949 955. (10.1007/s00701-018-3499-7) 29470721

[27] Critical Appraisal Skills Programme. CASP (cohort study checklist) [online]. https://casp-uk.net/casp-tools-checklists/. Accessed may 04, 2023; 2022.

[28] Higgins J Greens S . Cochrane Handbook for Systematic Reviews of Interventions. Version 5.1.0; updated March 2011. The Cochrane Collaboration:2011. Available at: www.cochrane-handbook.org. Accessed April 03, 2023.

[29] Szabo SM Levy AR Rao SR , et al. Increased risk of cardiovascular and cerebrovascular diseases in individuals with ankylosing spondylitis: A population‐based study. Arthritis Rheum. 2011;63(11):3294 3304. (10.1002/art.30581) 21834064

[30] Bremander A Petersson IF Bergman S Englund M . Population‐based estimates of common comorbidities and cardiovascular disease in ankylosing spondylitis. Arthritis Care Res. 2011;63(4):550 556. (10.1002/acr.20408) 21452267

[31] Zöller B Li X Sundquist J Sundquist K . Risk of subsequent ischemic and hemorrhagic stroke in patients hospitalized for immune-mediated diseases: a nationwide follow-up study from Sweden. BMC Neurol. 2012;12(1):41. (10.1186/1471-2377-12-41) 22708578 PMC3430565

[32] Keller JJ Hsu JL Lin SM , et al. Increased risk of stroke among patients with ankylosing spondylitis: a population-based matched-cohort study. Rheumatol Int. 2014;34(2):255 263. (10.1007/s00296-013-2912-z) 24322454

[33] Huang YP Wang YH Pan SL . Increased risk of ischemic heart disease in young patients with newly diagnosed ankylosing spondylitis–a population-based longitudinal follow-up study. PLoS One. 2013;8(5):e64155. (10.1371/journal.pone.0064155) 23691161 PMC3655062

[34] Chou CH Lin MC Peng CL , et al. A nationwide population-based retrospective cohort study: increased risk of acute coronary syndrome in patients with ankylosing spondylitis. Scand J Rheumatol. 2014;43(2):132 136. (10.3109/03009742.2013.822097) 24134400

[35] Lin CW Huang YP Chiu YH Ho YT Pan SL . Increased risk of ischemic stroke in young patients with ankylosing spondylitis: a population-based longitudinal follow-up study. PLOS ONE. 2014;9(4):e94027. (10.1371/journal.pone.0094027) 24714094 PMC3979725

[36] Ahmed N Prior JA Chen Y Hayward R Mallen CD Hider SL . Prevalence of cardiovascular-related comorbidity in ankylosing spondylitis, psoriatic arthritis and psoriasis in primary care: a matched retrospective cohort study. Clin Rheumatol. 2016;35(12):3069 3073. (10.1007/s10067-016-3362-2) 27485152

[37] Eriksson JK Jacobsson L Bengtsson K Askling J . Is ankylosing spondylitis a risk factor for cardiovascular disease, and how do these risks compare with those in rheumatoid arthritis? Ann Rheum Dis. 2017;76(2):364 370. (10.1136/annrheumdis-2016-209315) 27283333

[38] Essers I Stolwijk C Boonen A , et al. Ankylosing spondylitis and risk of ischaemic heart disease: a population-based cohort study. Ann Rheum Dis. 2016;75(1):203 209. (10.1136/annrheumdis-2014-206147) 25362044

[39] Buschiazzo EA Schneeberger EE Sommerfleck FA Ledesma C Citera G . Mortality in patients with ankylosing spondylitis in Argentina. Clin Rheumatol. 2016;35(9):2229 2233. (10.1007/s10067-016-3336-4) 27377455

[40] Hung YM Chang WP Wei JC-C Chou P Wang PY-P . Midlife ankylosing spondylitis increases the risk of cardiovascular diseases in males 5 years later: a national population-based study. Medicine. 2016;95(18):e3596. (10.1097/MD.0000000000003596) 27149491 PMC4863808

[41] Bengtsson K Forsblad-d’Elia H Lie E , et al. Are ankylosing spondylitis, psoriatic arthritis and undifferentiated spondyloarthritis associated with an increased risk of cardiovascular events? A prospective nationwide population-based cohort study. Arthritis Res Ther. 2017;19(1):102. (10.1186/s13075-017-1315-z) 28521824 PMC5437558

[42] Lee JL Sinnathurai P Buchbinder R Hill C Lassere M March L . Biologics and cardiovascular events in inflammatory arthritis: a prospective national cohort study. Arthritis Res Ther. 2018;20(1):171. (10.1186/s13075-018-1669-x) 30086795 PMC6081907

[43] Walsh JA Song X Kim G Park Y . Evaluation of the comorbidity burden in patients with ankylosing spondylitis using a large US administrative claims data set. Clin Rheumatol. 2018;37(7):1869 1878. (10.1007/s10067-018-4086-2) 29637483 PMC6006197

[44] Derakhshan MH Goodson NJ Packham JC , et al. Increased risk of hypertension associated with spondyloarthritis disease duration: results from the ASAS-COMOSPA study. J Rheumatol. 2019;46(7):701 709. (10.3899/jrheum.180538) 30647169

[45] Trömmer K Kostev K Jacob L Tanislav C . Increased incidence of stroke and transient ischemic attack in patients with rheumatoid arthritis and ankylosing spondylitis in Germany. Neuroepidemiology. 2021;55(2):162 170. (10.1159/000514889) 33789293

[46] Higgins J . Cochrane Handbook for Systematic Reviews of Interventions. Version 5.1.0 [updated March 2011]. The Cochrane Collaboration. Available at: http://www.cochrane-handbook org; 2011.

[47] Zhu W He X Cheng K , et al. Ankylosing spondylitis: etiology, pathogenesis, and treatments. Bone Res. 2019;7(1):22. (10.1038/s41413-019-0057-8) 31666997 PMC6804882

[48] Kim J Park E Park M Cho J Son MH . Clinical features of adolescents who visited the emergency department with chest discomfort: the importance of recognizing underlying medical conditions. Pediatr Emerg Med J. 2020;7(2):70 76. (10.22470/pemj.2020.00101)

[49] Wong K Gladman DD Husted J Long JA Farewell VT Long JA . Mortality studies in psoriatic arthritis. Results from a single outpatient clinic. I. Causes and risk of death. Arthritis Rheum. 1997;40(10):1868 1872. (10.1002/art.1780401021) 9336423

[50] Ali Y Tom BD Schentag CT Farewell VT Gladman DD . Improved survival in psoriatic arthritis with calendar time. Arthritis Rheum. 2007;56(8):2708 2714. (10.1002/art.22800) 17665458

[51] Buckley C Cavill C Taylor G , et al. Mortality in psoriatic arthritis–a single-center study from the UK. J Rheumatol. 2010;37(10):2141 2144. (10.3899/jrheum.100034) 20682670

[52] BARTELS S Gjertsen JE Frihagen F Rogmark C Utvåg SE . Low bone density and high morbidity in patients between 55 and 70 years with displaced femoral neck fractures: a case-control study of 50 patients vs 150 normal controls. BMC Musculoskelet Disord. 2019;20(1):371. (10.1186/s12891-019-2732-8) 31409337 PMC6692959

[53] Divecha H Sattar N Rumley A Cherry L Lowe GD Sturrock R . Cardiovascular risk parameters in men with ankylosing spondylitis in comparison with non-inflammatory control subjects: relevance of systemic inflammation. Clin Sci (Lond). 2005;109(2):171 176. (10.1042/CS20040326) 15801904

[54] Liew JW Ramiro S Gensler LS . Cardiovascular morbidity and mortality in ankylosing spondylitis and psoriatic arthritis. Best Pract Res Clin Rheumatol. 2018;32(3):369 389. (10.1016/j.berh.2019.01.002) 31171309

